# Bromide of Potassium in Convulsions and Dental Irritation

**Published:** 1874-06

**Authors:** 


### EDITORIAL, ETC.

---

*Bromide of Potassium in Convulsions and Dental Irritation.*—Prof. Lewis Smith, in a paper read before the N. Y. Academy of Medicine, speaks of this remedy having been employed with marked success in convulsions of Infants:

"Three cases were mentioned belonging to this class. In the first case ½ gr. doses of the remedy were administered every two hours. Recovery. In the second case, two grains were given every three hours. Recovery. In the third case ¾ of a grain was given every three hours. Recovery.

In cases of *trismus nascentium* the bromide is much inferior to hydrate of chloral, for occasionally a case of this affection recovers under the influence of chloral, but the bromide of potassium is of no special service.

Dental Irritation.—The remedy, in this class of cases, is so so salutary in its effects, that the gum-lancet need scarcely ever be resorted to. It is to be administered in doses proportionate to the age of the child, and repeated according to circumstances. The remedy is of service in the treatment of all local diseases except gastro-intestinal irritation and renal disease, where convulsions are threatening."

